# Complete chloroplast genome of *Baccaurea ramiflora* (phyllanthaceae), a promising underutilized species

**DOI:** 10.1080/23802359.2021.1997105

**Published:** 2021-11-11

**Authors:** Gang Hu, Hongping Wu, Zhonghua Zhang, Lei Li

**Affiliations:** a Ministry of Education Key Laboratory for Ecology of Tropical Islands, Key Laboratory of Tropical Animal and Plant Ecology of Hainan Province, College of Life Sciences, Hainan Normal University, Haikou, China; b College of Environmental and Life Sciences, Nanning Normal University, Nanning, China

**Keywords:** *Baccaurea ramiflora*, chloroplast genome, phylogenetic analysis

## Abstract

*Baccaurea ramiflora* is a high economic value for the exploitation and utilization of wild fruit tree species with edible and medicinal values in Southeast Asia. In this study, the complete chloroplast genome sequence of *B. ramiflora* was assembled and characterized. The chloroplast genome was 161,089 bp in length, consisting of a large single copy (LSC) of 89,515 bp and a small single copy (SSC) region of 18,826 bp, which were separated by a pair of 26,374 bp inverted repeat (IR) regions. The overall GC content was 36.71%. A total of 132 genes, including 84 protein-coding genes, 37 tRNA genes, and 8 rRNA genes were identified. A low intraspecies variation within *B. ramiflora* was found with 22 single nucleotide polymorphisms (SNPs) and 17 insertions and deletions (INDELs). Phylogenetic tree reconstructed by 14 chloroplast genomes revealed that *B. ramiflora* clusters together with species of *Phyllanthus*, *Glochidion*, and *Flueggea*.

*Baccaurea ramiflora* Lour. (Phyllanthaceae) is an evergreen wild fruit tree species that is native to Southeast Asia (India, Laos, Malaysia, Thailand, Vietnam, and south China), chiefly distributed in moist tropical forests (Tongkok et al. 2020). The fruit of this plant species has high utilization value because of its high content of vitamin C, protein, and minerals content (Amin and Nabi 2015; Goyal et al. 2020). Its leaves, fruits, stems, bark, and seeds form an ingredient in many herbal prescriptions which have been used to treat many human diseases, such as jaundice, constipation, indigestion, and cellulitis (Dutta et al. 2018; Uddin et al. 2018). Several previous studies were carried out to identify the chemical constituents and pharmacological activities of this plant (Goyal et al. 2020). Therefore, *B. ramiflora* is a promising underutilized tree species with edible and medicinal values in Southeast Asia. It is necessary to develop genomic resources for *B. ramiflora* to provide basic intragenic information for the further study on phylogeny and breeding for genus *Baccaurea*.

The fresh leaves of *B. ramiflora* were sampled from Nonggang National Nature Reserve, Guangxi Province, south China (N22°28′22″, E106°55′7″). The voucher specimen (NNNU 2020110308) was deposited in the herbarium of the College of Environmental and Life Sciences, Nanning Normal University, China. Total genomic DNA was extracted from the fresh leaves with a modified CTAB protocol according to Doyle and Doyle (1987). The complete genome sequencing was conducted by Genepioneer Biotechnologies Inc. (Nanjing, China) on the Illumina Hiseq platform. The filtered sequences were assembled using the program SPAdes assembler 3.10.0 (Bankevich et al. 2012). Annotation was performed using the DOGMA (Wyman et al. 2004) and BLAST searches.

The complete chloroplast genome of *B. ramiflora* was determined to comprise a 161,089 bp double-stranded, circular DNA (GenBank accession no. MW717296), which contains two inverted repeats (IR) regions of 26,374 bp, separated by large single-copy (LSC) and small single-copy (SSC) regions of 89,515 bp and 18,826 bp, respectively. The GC content of the chloroplast genome is 36.71%, meanwhile, the corresponding values in LSC, SSC, and IR regions are 34.41%, 34.99%, and 42.71%, respectively. The complete chloroplast genome was predicted to contain 132 genes, including 84 protein-coding genes, 37 tRNA genes, and 8 rRNA genes. 8 protein-coding genes, 7 tRNA genes, and 4 rRNA genes were duplicated in IR regions. Twenty genes contained one intron, while two genes had two introns.

To explore the divergence hotspot regions within *B. ramiflora*, the genome sequence was compared to another available *B. ramiflora* that was collected from Yunnan Province, south China (GenBank accession no. MT900598). A low-level molecular variation within *B. ramiflora* was found with 22 single nucleotide polymorphisms (SNPs) and 17 insertions and deletions (INDELs). There were three and one synonymous substitutions in *ycf2* and *psbI*, respectively, and one nonsynonymous change in *atpI*, *rpoC1,* respectively. The remaining 16 SNPs were located in the non-coding regions. Two of the 17 INDELs were found in the CDS region. The results indicated that the number of intraspecific variations of *B. ramiflora* between Guangxi and Yunan provinces of China is relatively small.

To investigate its taxonomic status, alignment was performed on the 14 chloroplast genome sequences using MAFFT v7.307 (Katoh and Standley 2013), and a maximum likelihood (ML) tree (*Ficus carica* and *Artocarpus heterophyllus* were used as the outgroups) was constructed based on complete chloroplast genomes by RAxML v8.2.10 (Stamatakis 2014). The results indicate that *B. ramiflora* clusters together with species of *Phyllanthus*, *Glochidion*, and *Flueggea* (Figure 1). The complete chloroplast genome sequence of *B. ramiflora* will provide a useful resource for the conservation genetics of this species as well as for the phylogenetic studies of Phyllanthaceae.

**Figure 1.**
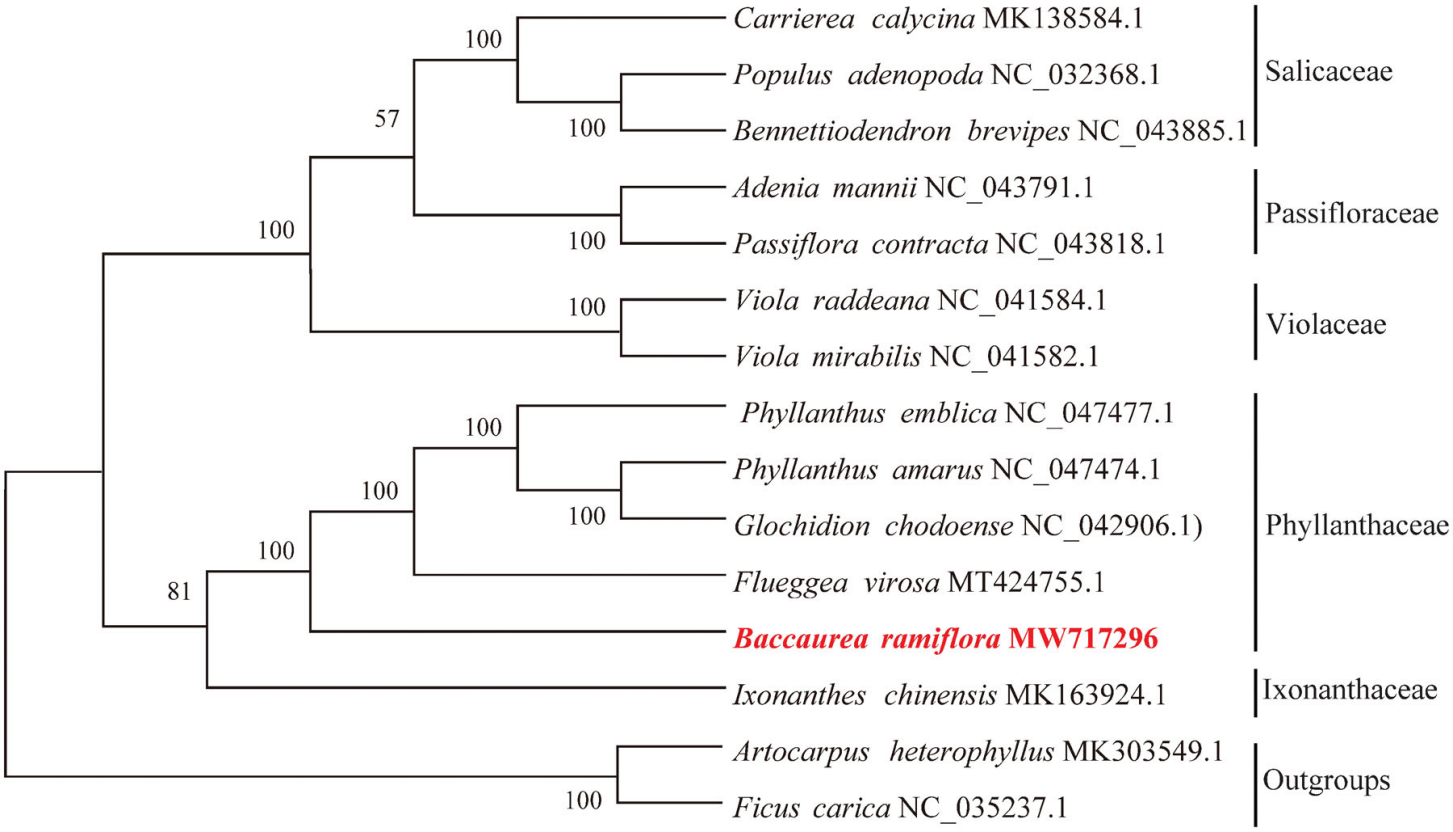
Phylogenetic relationship of the *B. ramiflora* chloroplast genome with 14 previously reported complete chloroplast genomes. A total of 1000 bootstrap replicates were computed and the bootstrap support values are shown at the branches.

## Data Availability

The genome sequence data that support the findings of this study are openly available in GenBank of NCBI at https://www.ncbi.nlm.nih.gov/nuccore/MW717296 under the accession NO. MW717296. The associated BioProject, SRA, and Bio-Sample numbers are PRJNA708152, SRR13907804, and SAMN18220041, respectively.
